# Supplementary material for: Content-rich biological network constructed by mining PubMed abstracts
Source: BMC Bioinformatics. 2004 Oct 8;5:147. doi: 10.1186/1471-2105-5-147 (PMC528731; doi:10.1186/1471-2105-5-147)
Supplement: Additional File 2 — The original results of the above study (non-essential files are deleted to keep the file size under the limit set by BMC bioinformatics). [file 1471-2105-5-147-S2.bz2 › chilibotAdditionalFile2/dip05/121ID8668348E465/html/YWHAQ_RAF1.html]

 


 **YWHAQ** and **RAF1** 
  
Found 1 abstracts in PubMed, retrieved 1.  
 

 What does Google say? 
 PDF only 
| .edu only 

---

- Nature, 1994   **Activation of Raf 1 **[RAF1]** by 14 3 3 proteins.** .
  The protein Raf 1 **[RAF1]**, a key mediator of mitogenesis and differentiation, associates with p21ras refs 1 3 .
  However, the regulation of the serine threonine kinase activity of Raf 1 **[RAF1]** is still not understood.
  Using the yeast two hybrid system, we identified two structurally related proteins that interact with the aminoterminal region of Raf 1 **[RAF1]**.
  These proteins, 14 3 3 zeta PLA2 and 14 3 3 beta HS1 **[YWHAQ]** , are members of the 14 3 3 family of proteins.
  Expression of 14 3 3 proteins in Xenopus oocytes enhanced Raf 1 **[RAF1]** activity and promoted Raf 1 **[RAF1]** dependent oocyte maturation.
  A dominant negative mutant of Raf 1 **[RAF1]** blocked the effects of 14 3 3 protein.
